# IFITM2 Presents Antiviral Response through Enhancing Type I IFN Signaling Pathway

**DOI:** 10.3390/v15040866

**Published:** 2023-03-28

**Authors:** Lei Chen, Xiangrong Li, Yingying Deng, Yingjie Bi, Zhenfang Yan, Yanmei Yang, Xiangbo Zhang, Huixia Li, Jingying Xie, Ruofei Feng

**Affiliations:** 1 Key Laboratory of Biotechnology and Bioengineering of State Ethnic Affairs Commission, Biomedical Research Center, Northwest Minzu University, Lanzhou 730030, China; 2 Gansu Tech Innovation Center of Animal Cell, Biomedical Research Center, Northwest Minzu University, Lanzhou 730030, China; 3 College of Life science and Engineering, Northwest Minzu University, Lanzhou 730030, China

**Keywords:** IFITM2 protein, type I interferon, antiviral effect, MDA5, innate immune response

## Abstract

Interferon (IFN) helps cells fight viral infections by further inducing the expression of many downstream IFN-stimulated genes (ISGs). Human interferon-inducible transmembrane proteins (IFITM) are one of these ISGs. The antiviral function of human IFITM1, IFITM2, and IFITM3 are well known. In this study, we report that IFITM can significantly inhibit EMCV infectivity in HEK293 cells. Overexpression of IFITM proteins could promote IFN-β production. Meanwhile, IFITMs facilitated type I IFN signaling pathway adaptor MDA5 expression. We detected the binding of IFITM2 to MDA5 in a co-immunoprecipitation assay. It was also found that the ability of IFITM2 to activate IFN-β was significantly inhibited after interfering with MDA5 expression, suggesting that MDA5 may play an important role in the activation of the IFN-β signaling pathway by IFITM2. Moreover, the N-terminal domain plays an active role in the antiviral activity and the activation of IFN-β by IFITM2. These findings suggest that IFITM2 plays a vital role in antiviral signaling transduction. In addition, a positive feed-forward loop between IFITM2 and type I IFN establishes a key role for IFITM2 in enforcing innate immune responses.

## 1. Introduction

Type I interferons (IFNs) are a major host defense against viruses [1]. A typical antiviral type I IFN response is characterized by the production of IFN-β, which usually occurs soon after viral infection. Type I IFN exerts its antiviral function by binding to its receptors and activating JAK-STAT signaling, which finally induces the expression of IFN-stimulated genes (ISGs) [2]. The production and downstream signaling of type I IFN is necessary for host innate antiviral immunity.

Restriction factors are a diverse group of host proteins united in the same goal of antagonizing viral replication. Multiple restriction mechanisms have evolved, with some factors having activity against one virus and others acting broadly across several viral families. The transcription expression of many restriction factors is controlled by the antiviral cytokine interferon (IFN). Type I IFNs (IFN-α/β) are major antiviral cytokines and can be induced by several different pattern recognition receptors, including Toll-like receptors and cytoplasmic RIG-I-like helicases [3]. After induction, type I IFNs signal through the type I IFN receptor (IFNAR) to induce many ISGs. Hundreds of ISGs have been identified, of which only a handful have a characterized function [4]. Some induce broad antiviral responses, such as an inhibition of cellular protein synthesis or degradation of RNA [5,6].

IFN-inducible transmembrane proteins (IFITMs) are a group of ISGs known to restrict a specific class of virus and are conserved across numerous vertebrate species. In humans, the IFITM family contains five functional genes (IFITM1, IFITM2, IFITM3, IFITM5, and IFITM10). Among these, IFITM1, IFITM2, and IFITM3 are constitutively expressed in many tissues and can be induced by type I and II IFNs. To date, IFITM proteins 1, 2, and 3 have been shown to restrict viral entry and infection of several pathogenic enveloped viruses, including influenza A virus (IAV) [7], West Nile virus (WNV) [8], dengue virus (DENV) [9], Ebola virus (EBOV) [10], SARS coronavirus [11], rift valley fever virus [12], Semliki forest virus [13], human immunodeficiency virus type-1 (HIV-1) [14,15], respiratory syncytial virus (RSV) [16], and Zika virus [17]. However, the transcriptional regulation of IFITM proteins, especially in initiating type I IFN induction, remains unclear. In the previous research, we found that IFITM proteins had a significant inhibitory effect on PRV infection, which belonged to DNA virus, so we wanted to clarify the effect of IFITM proteins on RNA virus infection. Considering that the current research group is devoted to the study of picornaviruses, we chose EMCV as the model of virus infection to study the effect of IFITM proteins on the proliferation of EMCV and explore the relationship between IFITM proteins and type I IFN signaling pathway. We hypothesize that restriction of viral infectivity by IFITM proteins is a sustained process requiring type I IFNs to be continually activated while controlling the infection. Hence, IFITM could act as a previously uncharacterized positive regulator for type I IFN induction during a viral infection.

We show that IFITM is required for type I IFN induction and interacts with microbial RNA sensing machinery components to enhance IFN-β production further. These findings expand our knowledge of the mechanisms underlying the transcriptional regulation of IFITM2 expression and its ability to mediate the IFN signaling pathway. Thus, our results characterized IFITM proteins as an important modulator in the antiviral innate immune response.

## 2. Materials and Methods

### 2.1. Plasmids

All plasmids needed in this study were generated and preserved in our laboratory. Fragments encoding human IFITM1, IFITM2, and IFITM3 were generated from HEK293 cDNA and utilized specific primers. After digestion and extraction, genes were cloned into expression plasmid pCMV-Myc. The primer sequences used in this study are available upon request. All plasmids were verified using sequencing.

### 2.2. Cells, Viruses, and Reagents

Human embryonic kidney cell line (HEK293) and Baby hamster kidney cell line (BHK-21) cells were maintained in Dulbecco’s Modified Eagle’s Medium (DMEM) (Minhai Bio, Lanzhou, China). All cells were cultivated in a medium supplemented with 10% Fetal Bovine Serum (FBS) (Hyclone, Logan, UT, USA) at 37 °C 5% CO_2_. EMCV PV21 strain was preserved and provided by the Biomedical Research Center of Northwest Minzu University. Viruses were propagated in the BHK-21 cells. Viral stocks were stored at −80 °C and titrated on BHK-21 cells.

Recombinant Human IFN-α2a was purchased from 3SBIO INC (Shenyang, China). PowerUp™ SYBR™ Green Master Mix was from Applied Biosystems™ (Carlsbad, CA, USA). Rabbit polyclonal antibodies specific for IFITM2 (12769-1-AP) and IFITM3 (11714-1-AP), Mouse monoclonal antibodies specific for IFITM1 (60074-1-Ig), IFIH1/MDA5 Polyclonal antibody (21775-1-AP), MAVS Polyclonal antibody (14341-1-AP), and IRF3 Polyclonal antibody (11312-1-AP) were purchased from Proteintech (Wuhan, China). Anti-FLAG tag rabbit polyclonal antibody (D110005), HRP-conjugated Goat Anti-Rabbit IgG (D110058), and HRP-conjugated Goat Anti-Mouse IgG (D110087) were purchased from Sangon Biotech (Shanghai, China). TBK1 antibody (3013S) and Myc-Tag (9B11) Mouse mAb (2276S) were bought from Cell Signaling Technology. Mouse anti-Rab5 and Rab7 monoclonal antibodies were bought from Santa Cruz Biotechnology (Santa Cruz, CA, USA). GAPDH Mouse Monoclonal antibody (AF5009), β-actin Mouse Monoclonal antibody (AA128) and Caveolin-1 Rabbit Monoclonal antibody (AF1231) were purchased from Beyotime Biotechnology (Shanghai, China). Lipofectamine 2000 was purchased from Invitrogen (Carlsbad, CA, USA).

### 2.3. Transfection

HEK293 cells, at a confluence of 80% in a 6-well plate, were transfected with indicated plasmids according to the manufacturer’s instructions with Lipofectamine 2000 reagent. Cell-free culture supernatants and cell lysates were harvested at indicated time points and stored at −80 °C until use.

### 2.4. Evaluation of the Inhibitory Effect of IFITM Proteins on EMCV Infection

Viral infection was performed at 24 h post IFITM plasmids transfection. Cells were infected with EMCV at a multiplicity of infection (MOI) of 0.001 and cultured at 37 °C. The medium was removed 2 h later, and cells were cultured in DMEM containing 3% FBS.

### 2.5. RNA Extraction and Quantitative RT-PCR (RT-qPCR)

Total RNA was extracted with RNAiso Plus (TaKaRa, Dalian, China) according to the manufacturer’s instructions. For the first-strand cDNA synthesis, 1 μg of total RNA was reverse transcribed using Oligo(dT)_18_ Primer. RT-qPCR reactions were carried out using PowerUp™ SYBR™ Green Master Mix and performed in an ABI PRISM 7500 cycler (Applied Biosystems, Carlsbad, CA, USA). All values were normalized to the level of β-actin mRNA. All experiments were performed using the following amplification profile: one cycle at 94 °C for 30 s, 40 cycles at 94 °C for 5 s, and 60 °C for 34 s. As described previously, viral RNA copies in EMCV-infected cells were detected using TaqMan real-time PCR. Primers for RT-qPCR are available upon request.

### 2.6. RNAi Assay

siRNA targets MDA5 and MAVS were designed and synthesized by RiboBio (Guangzhou, China). siRNA transfection was performed using Lipofectamine 2000 following the manufacturer’s instructions. In brief, HEK293 cells were seeded in 6-well plates. When cells grew at a confluence of 50%, they were transfected with 150 nM siRNA using Lipofectamine 2000. The efficiency of MDA5 or MAVS expression downregulation was confirmed via Western blotting at 24 h post-transfection.

### 2.7. Western Blotting

Cells were lysed in a RIPA lysis buffer containing protease inhibitors. Cellular debris was removed from the samples via centrifugation at 10,000× *g* for 20 min at 4 °C. The clarified supernatant was resuspended in a 5× SDS sample buffer, boiled (5 min at 95 °C), and then electrophoresed on a 15% or 10% SDS-PAGE gel at 70 V for 90 min in a Tris–Glycine running buffer. Bands were transferred to a polyvinylidene difluoride (PVDF) membrane (Bio-rad, Hercules, CA, USA) via the semi-dry transfer method. After incubating in a blocking buffer (2.5% skimmed milk in phosphate-buffered saline with Tween 20) at room temperature for 1 h, the samples were probed with the indicated specific primary antibodies corresponding to HRP-conjugated IgG secondary antibodies. The samples were reacted using enhanced chemiluminescent (ECL) substrates.

### 2.8. Virus Binding and Entry Assay

IFITM overexpression cells were used for EMCV binding and entry assay. The binding assay was performed in 6-well plates at 4 °C. HEK293 cells were overexpressed IFITM proteins for 24 h, then cells were infected with 0.001MOI EMCV. After two hours on ice, cells were washed with ice-cold PBS to remove unbound virus, and samples to measure bound virus were collected by scraping cells into PBS, followed by additional washing. For the virus entry study, first binding and washing at 4 °C then incubating samples at 37 °C for two hours in DMEM medium. Cells were then washed with PBS and treated with 0.05% trypsin and washed to remove surface bound virus. Samples were kept on ice after the final washing step, then processed for RNA. Additionally, typically RNase treatment was also performed prior to the RNA extraction and quantitation. Primers specific to the EMCV genomic RNA detection were used for RT-qPCR.

### 2.9. Co-Immunoprecipitation Assay

Cells were collected with a lysis buffer supplemented with a protease inhibitor cocktail and incubated with the anti-Myc or anti-FLAG antibody for 12 h at 4 °C. Then, 10 μL of Protein G agarose slurry (Beyotime, Shanghai, China) was added to each lysate. After incubation for 4 h at 4 °C, the lysates were centrifuged at 2500 rpm for 5 min. The beads were collected and washed 5 times with ice-cold PBS. The precipitates were mixed with an SDS buffer and boiled for 5 min at 95 °C. After centrifugation at 6000 rpm for 1 min, the supernatant was collected and used for Western blot analysis.

### 2.10. ELISA

The concentration of IFN-β production was measured using ELISA kits according to the manufacturer’s instructions (Elabscience, Wuhan, China).

### 2.11. Statistical Analysis

Statistical significance was determined with one-way analysis of variance (ANOVA) or Student’s *t*-test. Values were expressed in graph bars as mean ± SDs of at least three independent experiments unless otherwise noted. Asterisks denote statistically significant differences (*** *p* < 0.001, ** *p* < 0.01, and * *p* < 0.05).

## 3. Results

### 3.1. IFITM Proteins Are Crucial for Controlling EMCV Infectivity

**Figure 1:**
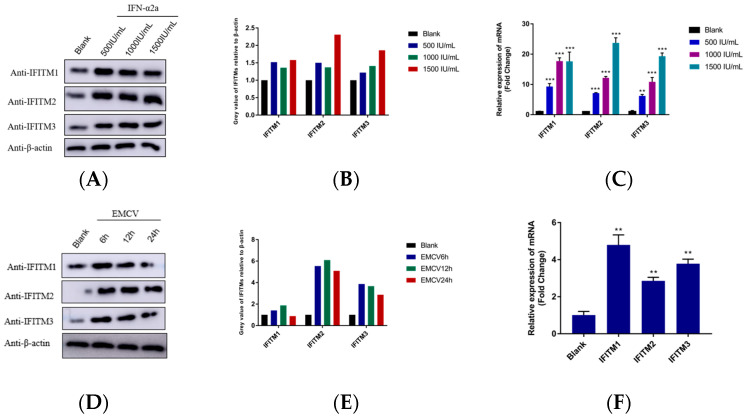
(**A**) HEK293 cells were cultured in 6-well plates. When cells grew to a dense monolayer, cells were treated with different concentrations of IFNα-2a (0, 500, 1000, 1500 IU/mL) for 12 h. Cells were collected and lysis with RIPA for IFITM proteins detection with a specific antibody. β-actin served as an internal control. (**B**) Greyscale analysis was used to measure the protein expression level of IFITM proteins relative to β-actin. (**C**) HEK293 cells were cultured in 6-well plates. When cells grew to a dense monolayer, cells were treated with different concentrations of IFNα-2a (0, 500, 1000, 1500 IU/mL) for 12 h. Cells were collected, and cellular RNA was extracted for IFITMs mRNA detection via RT-qPCR. (**D**) HEK293 cells were cultured in 6-well plates. When cells grew to a dense monolayer, cells were infected with 0.001 MOI EMCV for 6, 12, and 24 h. Cells were collected and lysis with RIPA for IFITM proteins detection with a specific antibody. β-actin served as a loading control. (**E**) Greyscale analysis was used to measure the protein expression level of IFITM proteins relative to β-actin. (**F**) HEK293 cells were cultured in 6-well plates. When cells grew to a dense monolayer, cells were infected with 0.001 MOI EMCV for 24 h. Cells were collected, and cellular RNA was extracted for IFITMs’ mRNA detection via RT-qPCR. Data were expressed as mean ± SDs from three independent experiments and were measured in technical duplicate. Comparisons between groups were performed using the Student’s *t*-test. *** *p* < 0.001, ** *p* < 0.01.

To assess the inducibility of IFITMs in HEK293 cells, we evaluated the expression of IFITM in the presence of recombinant IFNα-2a. Indeed, IFN-α2a could significantly induce IFITM1/2/3 expression in HEK293 cells (Figure 1A–C). Then, the expression of IFITM1/2/3 during EMCV infection was also detected. As shown in Figure 1D–F, there was an increased expression of IFITMs in HEK293 cells. We also noticed the observed trend of the Western blotting result and the mRNA relative expression level at 24 h was not consistent. The possible reasons are that the expression of protein and genes is sequential, or that the protein encoded by EMCV can inhibit the expression of IFITM genes after virus infection, but the specific reasons need further experimental analysis. The only thing we know for sure is that EMCV infection upregulates the expression of IFITM proteins.

**Figure 2:**
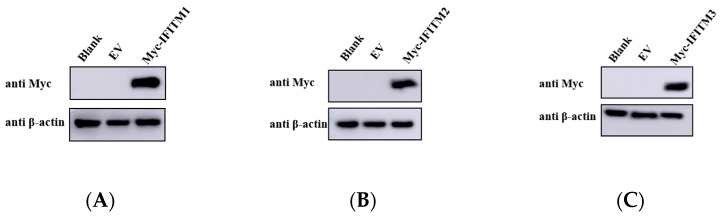

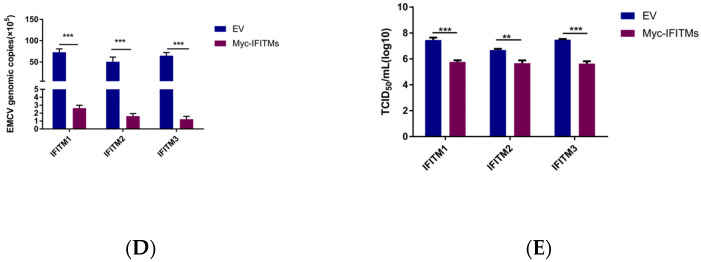
HEK293 cells were transfected with pCMV-Myc-IFITM1 (1 μg), -IFITM2 (1 μg), and -IFITM3 (1 μg) plasmid, respectively. pCMV-Myc empty vector (1 μg) served as control. Cells were infected 24 h post-transfection with 0.001 MOI EMCV PV21 strain for 24 h. Then, collected virus for viral genomic copies detection or determining infectious progeny viral titers via the TCID_50_ assay. (**A**–**C**) IFITM proteins overexpression was detected via Western blotting in HEK293 cells with a Myc-tagged antibody. β-actin served as a loading control. (**D**) EMCV genomic RNA copies were detected using real-time TaqMan PCR. (**E**) Infectious progeny viral titers were determined via the TCID_50_ assay. Data were represented as mean ± SDs of three independent experiments and were measured in technical duplication. Comparisons between groups were performed using the Student’s *t*-test. *** *p* < 0.001, ** *p* < 0.01.

Next, we wanted to investigate the impact of IFITM proteins have on viral replication. We used the encephalomyocarditis virus (EMCV) as a viral infection model. HEK293 cells transiently overexpressing IFITM1, IFITM2, or IFITM3 (Figure 2A–C) were infected with EMCV. Compared to cells transfected with the empty plasmid, EMCV genomic copy number (Figure 2D) and titer (Figure 2E) were significantly lower in cells overexpressing IFITM proteins, suggesting that EMCV replication was restricted by the presence of IFITM proteins.

**Figure 3:**
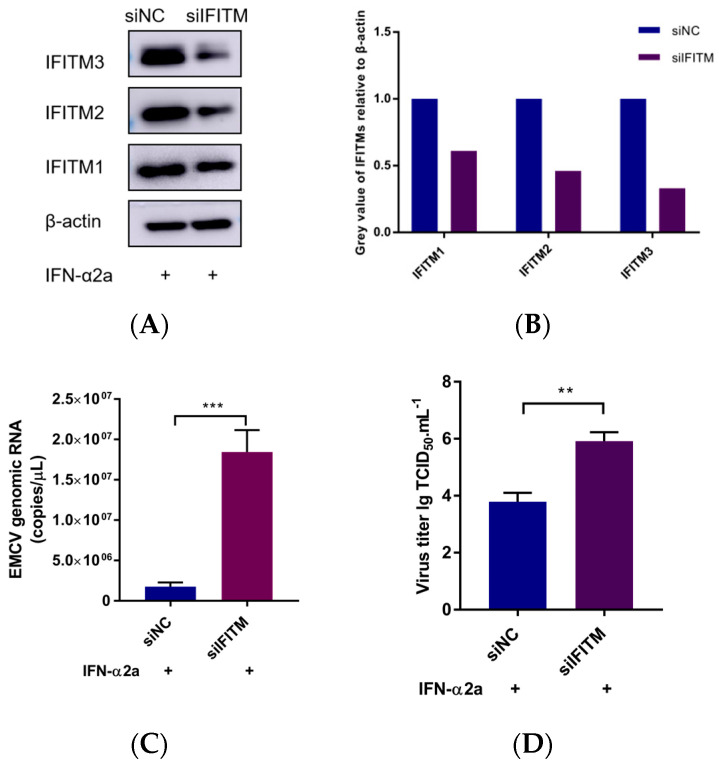
(**A**) HEK293 cells were pretreated with IFN-α2a 1000 IU/mL for 12 h and transfected with 150 nM siRNA targeting IFITMs proteins. After 24 h, IFITM proteins expression were analyzed via Western blotting using specific antibodies. β-actin served as the loading control. (**B**) Greyscale analysis was used to measure the protein expression level of IFITM proteins relative to β-actin. (**C**) Cells with knocked down IFITMs were infected with 0.001 MOI EMCV for 24 h, the virus was collected, and viral RNA was extracted for EMCV genomic RNA copies detection via real-time TaqMan PCR. (**D**) Infectious progeny viral titers were determined via the TCID_50_ assay. Data were expressed as mean ± SDs from three independent experiments and were measured in technical duplicate. Comparisons between groups were performed using the Student’s *t*-test. *** *p* < 0.001, ** *p* < 0.01.

To evaluate the role of IFITM in EMCV replication, HEK293 cells were transfected with siRNA for 24 h. As IFITM1, IFITM2, and IFITM3 have high amino acid similarity, we designed a set of siRNAs targeting IFITM1, IFITM2, and IFITM3. Loss of IFITM family members’ protein expression by siRNA knockdown (Figure 3A,B) significantly increased the EMCV viral copies’ number and titer (Figure 3C,D). We show that IFITM is crucial for preventing EMCV replication and infectivity.

### 3.2. IFITM Proteins Enhanced IFN-β Production during EMCV Infection

**Figure 4:**
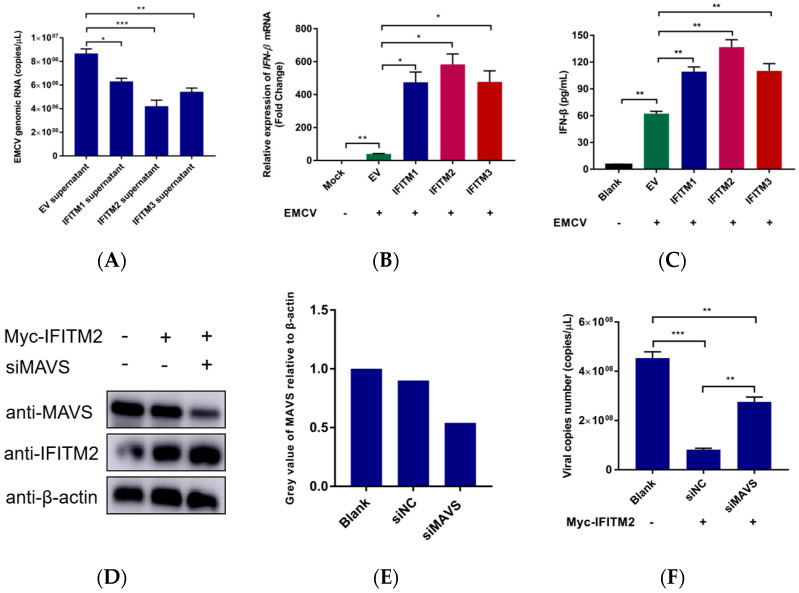
HEK293 cells were transfected with Myc-IFITM1 (1 μg), -IFITM2 (1 μg), or -IFITM3 (1 μg) plasmid, respectively. pCMV-Myc empty vector (1 μg) served as control. (**A**) Cell culture supernatant was collected 24 h post-transfection and added to EMCV infection cells, serving as a sustainable medium. Virus-infected cells were cultured for another 24 h, then collected for viral RNA extraction. Viral genomic copies were detected using real-time PCR using specific primers. (**B**) 24 h post-transfection, cells were infected with 0.001 MOI EMCV for 12 h. Cellular RNA was extracted for IFN-β mRNA detection via RT-qPCR. Data were normalized to β-actin expression. (**C**) IFN-β concentrations in the culture supernatant of overexpression IFITM proteins were detected via ELISA according to the manufacturer’s instructions. Data were expressed as mean ± SDs from three independent experiments and were measured in technical duplicate. Comparisons between groups were performed using the Student’s *t*-test. *** *p* < 0.001, ** *p* < 0.01, * *p* < 0.05. (**D**) HEK293 cells were transfected with or without siRNA-targeting MAVS for 24 h, followed by IFITM2 overexpression for another 24 h. Then, cells were collected for MAVS and IFITM2 detection used specific antibodies. β-actin served as the loading control. (**E**) Greyscale analysis was used to measure the protein expression level of MAVS proteins relative to β-actin. (**F**) HEK293 cells were transfected with siRNA NC or siRNA-targeting MAVS for 24 h, followed by IFITM2 overexpression for another 24 h. Then, cells were infected with 0.01 MOI EMCV for 24 h. Virus was collected for RNA extraction and viral copies number was detected via real-time TaqMan PCR. Data were expressed as mean ± SDs from three independent experiments and were measured in technical duplicate. Comparisons between groups were performed using the Student’s *t*-test. *** *p* < 0.001, ** *p* < 0.01.

We hypothesized that decreased EMCV infectivity was due to IFN-β being released into the cell media of IFITM overexpressing cells and working in an autocrine manner to dampen EMCV infectivity. To test this hypothesis, parental HEK293 cells were infected with EMCV, exposing cells to cell culture media from IFITM overexpressing cells for 24 h. Remarkably, the number of EMCV copies was significantly reduced (Figure 4A). Indeed, we also detected the production and secretion of IFN-β in IFITM overexpression cells during EMCV infection, and results showed IFITM proteins enhanced EMCV-triggered IFN-β production (Figure 4B,C). These results suggest that the type I IFN signaling pathway is active in these cells.

To further verify the above hypothesis, we performed a MAVS interference experiment. As MAVS is a key linker molecule of IFN signaling pathway, we performed IFITM2 overexpression in the context of RNAi MAVS expression, then examined the ability of IFITM2 on restriction EMCV replication at the same time. Interestingly, we found that there was a significant increase in the genomic copies number of EMCV in the presence of knockdown MAVS expression (Figure 4D–F). All together we can clearly conclude that IFITM2 restricted EMCV infection in HEK293 cells dependent on the secretion expression of IFN-β.

**Figure 5:**
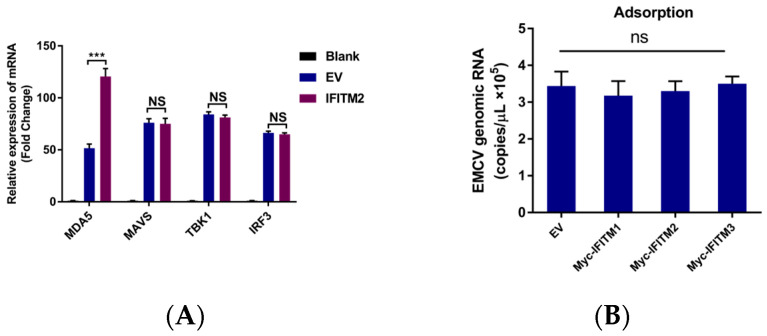

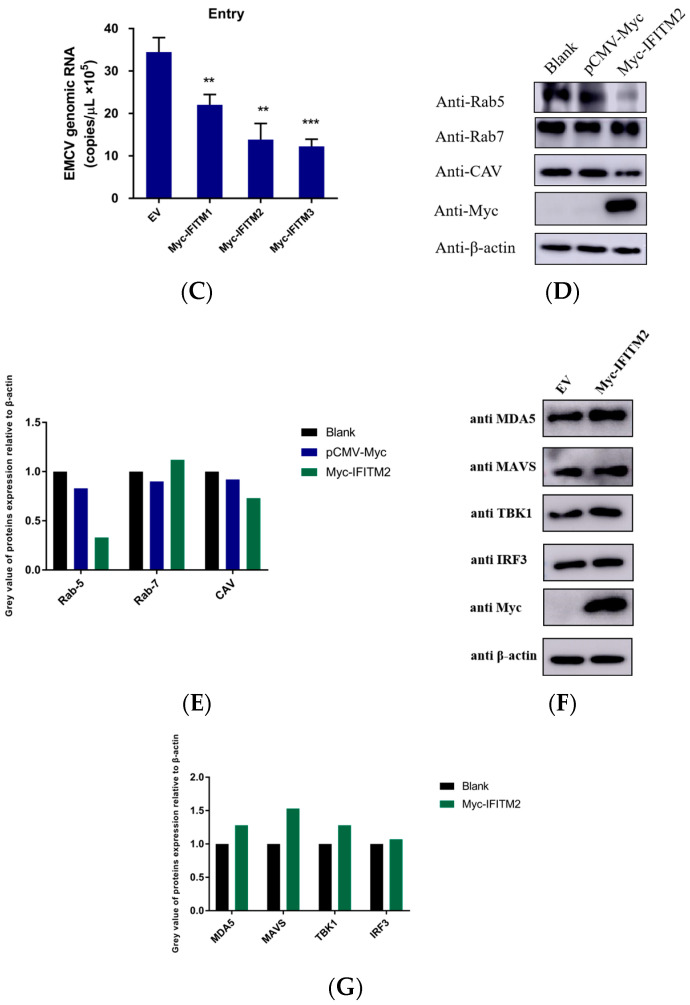
(**A**) HEK293 cells were transfected with pCMV-MyC-IFITM2 (1 μg) and pCMV-Myc (1 μg), respectively. Cells were stimulated 24 h post-transfection with 0.001 MOI EMCV for another 12 h. Then, cells were collected, and RNA was extracted for MDA5, MAVS, TBK1, and IRF3 detection via RT-qPCR. All values were normalized to the level of β-actin mRNA. Data were expressed as mean ± SDs from three independent experiments and were measured in technical duplicate. Comparisons between groups were performed using the Student’s *t*-test. *** *p* < 0.001. (**B**) HEK293 cells overexpression of IFITM proteins were used for the viral adsorption experiment. Cells were infected with EMCV at an MOI of 0.001. Then, cells were incubated at 4 °C for 2 h, washed 4 times with cold PBS, and collected for RNA extraction. real-time PCR was applied to detect virus copies bound to the cell surface. (**C**) HEK293 cells overexpression of IFITM proteins was used for viral entry assay. Cells were infected with EMCV at an MOI of 0.001. Then, cells were incubated at 4 °C for 2 h, washed 4 times with cold PBS, incubated at 37 °C for another 2 h, then cells were washed with PBS and treated with 0.05% trypsin, washed 4 times with cold PBS, and collected for RNA extraction. The viral entry into cells was detected via real-time TaqMan PCR. Data were expressed as mean ± SDs from three independent experiments and were measured in technical duplicate. Comparisons between groups were performed using the Student’s *t*-test. *** *p* < 0.001, ** *p* < 0.01. (**D**) HEK293 cells were transfected with pCMV-MyC-IFITM2 (1 μg), and cells transfected with pCMV-Myc (1 μg) served as control. Cells were collected 24 h post-transfection, and cellular protein was extracted for Rab5, Rab7, and Caveolin-1 detection via Western blotting. β-actin served as a loading control. (**E**) Greyscale analysis was used to measure the protein expression level relative to β-actin. (**F**) HEK293 cells were transfected with pCMV-MyC-IFITM2 (1 μg), and cells transfected with pCMV-Myc (1 μg) served as control. Cells were collected 24 h post-transfection, and cellular protein was extracted for MDA5, MAVS, TBK1, and IRF3 detection via Western blotting. β-actin served as a loading control. (**G**) Greyscale analysis was used to measure the protein expression level relative to β-actin.

To investigate the function of IFITMs, we wanted to determine the factors in the type I IFN signaling pathway which could trigger IFN-β expression. As shown in Figure 4, among these three IFITM proteins, IFITM2 showed the strongest antiviral effect and the ability to activate IFN-β. Based on this, we chose IFITM2 for further study. In IFITM2 overexpressed cells along with EMCV infection, IFITM2 only promoted melanoma differentiation-associated protein 5 (MDA5) expression at an mRNA level, but had no effect on its downstream signaling effector, MAVS, TBK1, and IRF3 (Figure 5A).

Based on these findings, we propose the hypothesis that IFITM can directly influence the process of viral adsorption and entry to inhibit viral replication or infection, and in addition, IFITM may also enhance its antiviral effects by enhancing the IFN-β signaling pathway. A series of studies indicated that the IFITM protein, through inhibiting virus entry, presents an antiviral role [6,7,8,9,10,11,12]. We also examined whether EMCV attachment and entry could be obstructed by the overexpression of IFITM proteins in HEK293 cells. Results showed that IFITM proteins did not affect EMCV adsorption but interfered with EMCV cell entry (Figure 5B,C).

A previous study had shown that EMCV infection is associated with the caveolar/lipid raft-dependent endocytic pathway [18]. In addition, both Rab5 and Rab7 are required for endocytosis. Given that the endocytic pathway could mediate IFITM restriction of viral infection, we hypothesized that overexpressed IFITM2 might affect endosomal compartments to interfere with EMCV infection in HEK293 cells. Western blotting analysis showed that Rab5 and Caveolin–1 (CAV) were decreased in IFITM2 overexpression cells (Figure 5D,E). Therefore, these results revealed that IFITM2 inhibited the EMCV entry process and affected the expression of endocytic pathway-related proteins Rab5 and Caveolin–1.

Figure 5A showed that overexpression of IFITM enhances the gene transcription levels of MDA5. Here, we further investigated the effect of the overexpression of IFITM2 on the protein expression levels of these factors during EMCV infection. As shown in Figure 5F,G, the protein expression levels of MDA5 have significantly enhanced in IFITM2 overexpressing cells. Therefore, we suspect that in addition to directly inhibiting the entry of the virus, IFITM2 also enhances its antiviral effect by enhancing IFN-β expression.

### 3.3. MDA5 Plays a Significant Role in IFITM2-Induced IFN-β Activation

**Figure 6:**
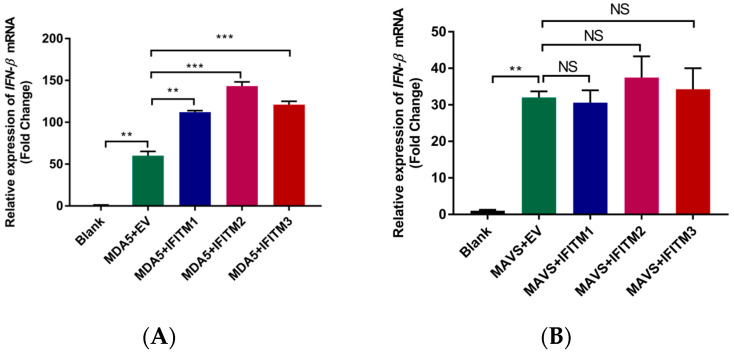

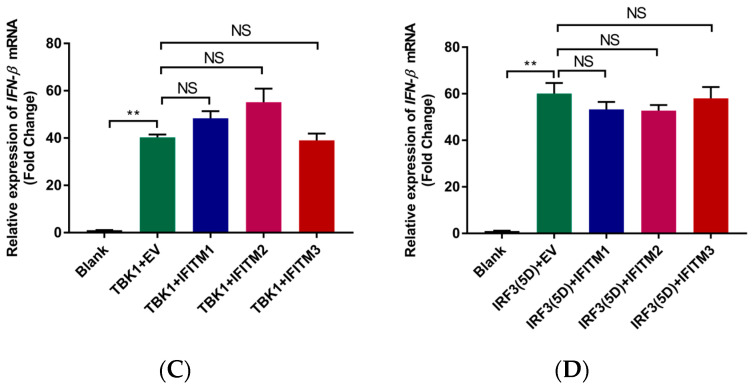
HEK293 cells were cotransfected with empty vector (0.5 μg) or Myc-IFITM1/-IFITM2/-IFITM3 (0.5 μg) plasmids and the indicated plasmids expressing MDA5 (0.2 μg) (**A**), MAVS (0.2 μg) (**B**), TBK1 (0.2 μg) (**C**), or IRF3(5D) (0.2 μg) (**D**) for 24 h. Then, cells were collected for total RNA extraction. IFN-β mRNA expression level was measured via RT-qPCR. Data were expressed as mean ± SDs from three independent experiments and were measured in technical duplicate. Comparisons between groups were performed using the Student’s *t*-test. *** *p* < 0.001, ** *p* < 0.01.

Although various cytokines and chemokines are produced by host cells, type I IFNs are the principal cytokines involved in antiviral response. To explore the mechanism of IFITM2 protein action, an RT-qPCR assay was performed, and the results are shown in Figure 6. IFITM2 could promote MDA5-triggered IFN-β activation (Figure 6A). However, IFITM2 did not affect IFN-β activation induced by MAVS, TBK1, and IRF3 (5D) (Figure 6B–D). These data reveal that MDA5 might be an IFITM2 function target to enhance the IFN-β signaling pathway.

**Figure 7:**
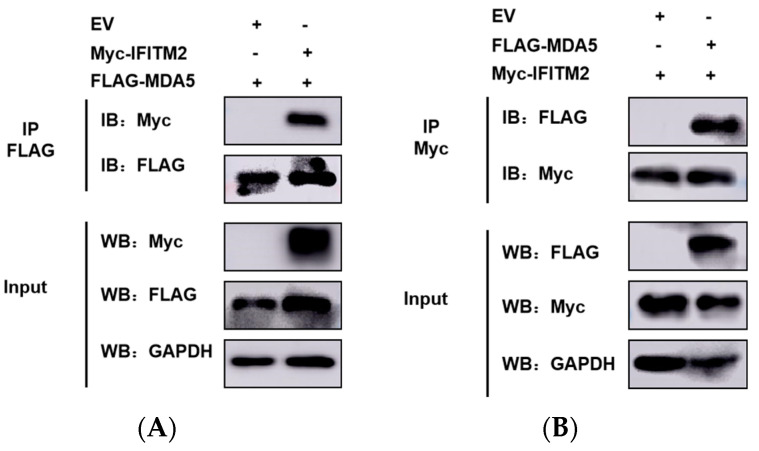
HEK293 cells were transfected with pCMV-MyC-IFITM2 (1 μg) or pCMV-Myc empty vector (EV, 1 μg) together with FLAG-MDA5 (0.5 μg) for 30 h before performing co-immunoprecipitations and immunoblotting with either anti-FLAG (**A**) or anti-Myc (**B**) antibodies. Anti-FLAG or anti-Myc antibody was used to incubate protein G agarose. The precipitates were mixed with SDS buffer and boiled for 5 min at 95 °C. After centrifugation at 6000 rpm for 1 min, the supernatant was collected and used for FLAG-tagged MDA5 and Myc-tagged IFITM2 analysis.

MDA5 plays an extremely important role in the induction of IFN response to EMCV infection. IFITM2 protein showed a significant effect on MDA5 expression and triggered IFN-β activation, suggesting that it could target MDA5 to enhance host innate immune responses. Given this, we did Co-IP to verify whether IFITM2 promoted IFN-β expression associated with the interaction between IFITM2 and MDA5. MyC-IFITM2 was co-transfected into HEK293 cells with FLAG-MDA5. Cell lysates were immunoprecipitated with anti-FLAG or anti-Myc antibodies. It was shown that IFITM2 could interact with MDA5 (Figure 7A,B). These data indicated that IFITM2 could interact with the host MDA5 protein.

**Figure 8:**
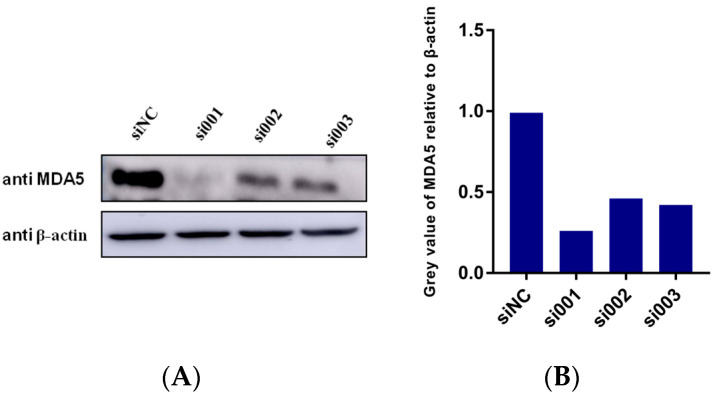

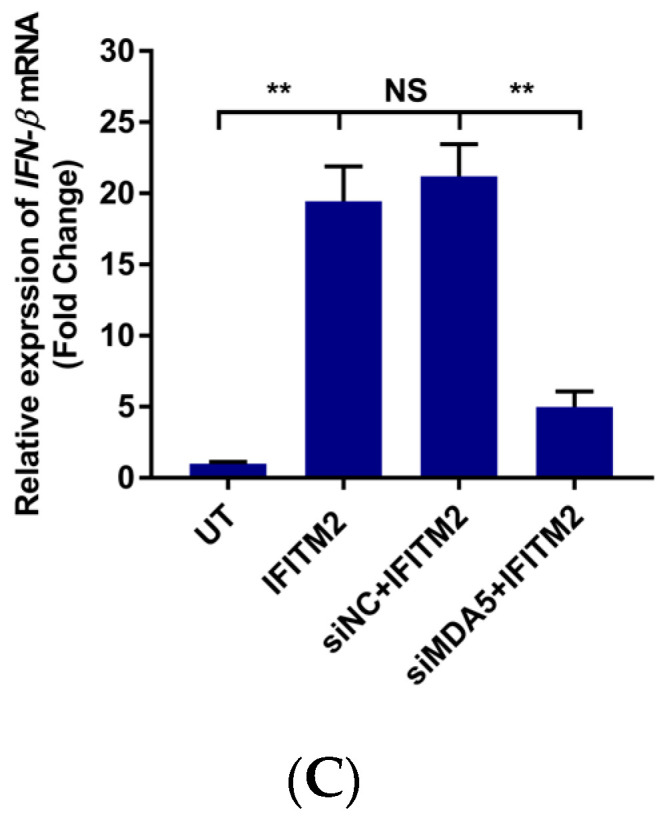
(**A**) 150 nM siRNA targeting MDA5 was transfected into HEK293 cells. Cells were collected 24 h post-transfection,. Cellular protein was extracted via RIPA and subjected to SDS-PAGE. Western blotting was performed for MDA5 expression by using a specific antibody. β-actin served as a loading control. (**B**) Greyscale analysis was used to measure the MDA5 expression level relative to β-actin. (**C**) HEK293 cells were transfected with 150 nM MDA5 siRNA. This was performed 24 h post-transfection, followed by transfecting with pCMV-MyC-IFITM2 (1 μg) for another 24 h. Cells were collected for cellular total RNA extraction. RT-qPCR was performed for IFN-β mRNA detection. All values were normalized to the level of β-actin mRNA. Data were expressed as mean ± SDs from three independent experiments and were measured in technical duplicate. Comparisons between groups were performed using the Student’s *t*-test. ** *p* < 0.01.

To determine if MDA5 plays a decisive role in IFITM2 activation of IFN-β, we designed and synthesized siRNAs targeting MDA5. The role of IFITM2 in the activation of IFN-β was verified by interfering with the expression of MDA5. Western blotting was performed for the knockdown of MDA5 detection. As shown in Figure 8A,B, the protein expression level of MDA5 decreased significantly in the presence of targeting siRNAs. Then, we analyzed the IFN-β mRNA transcription via RT-qPCR. Interestingly, we found that RNAi MDA5, IFITM2-induced IFN-β activation was decreased (Figure 8C). All these results indicated that MDA5 plays an essential role in IFITM2-induced IFN-β transcription.

### 3.4. IFITM2 N-terminal Domain, but C-terminal Domain, Involved in Activating IFN-β Transcription

**Figure 9:**
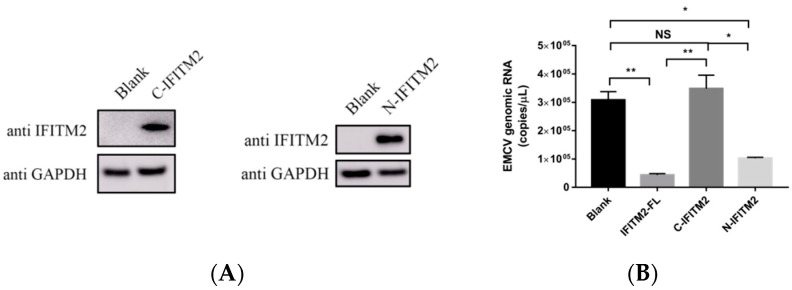

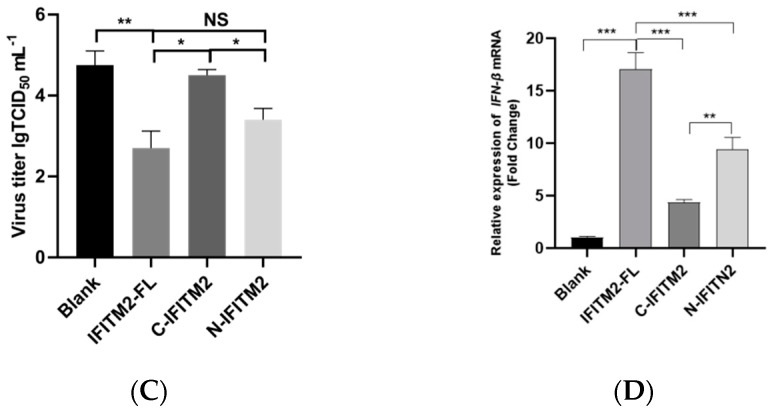
(**A**) HEK293 cells were cultured in 6-well plates when cells grew to a dense monolayer; transfection was performed according to the manufacturer’s instructions. IFITM2 truncated expression of plasmid C-terminal IFITM2 (1 μg), and N-terminal IFITM2 (1 μg) were transfected into HEK293 cells. Cells were collected 24 h later for IFITM2 detection. GAPDH served as a loading control. (**B**) HEK293 cells were transfected with the full length of IFITM2 (IFITM2-FL) (1 μg), N-terminal IFITM2 (N-IFITM2) (1 μg), and C-terminal IFITM2 (C-IFITM2) (1 μg) plasmid, respectively. Blank HEK293 cells served as control. Cells were infected 24 h post-transfection with 0.001 MOI EMCV PV21 strain for 24 h. Then, the virus was collected for viral genomic copies detection using real-time TaqMan PCR. (**C**) HEK293 cells were transfected with the full length of IFITM2 (IFITM2-FL) (1 μg), N-terminal IFITM2 (N-IFITM2) (1 μg), and C-terminal IFITM2 (C-IFITM2) (1 μg) plasmid, respectively. Blank HEK293 cells served as control. Cells were infected 24 h post-transfection with 0.001 MOI EMCV PV21 strain for 24 h. Then, the virus was collected, and infectious progeny viral titers were determined using the TCID_50_ assay. (**D**) HEK293 cells were transfected with the full length of IFITM2 (IFITM2-FL) (1 μg), N-terminal IFITM2 (N-IFITM2) (1 μg), and C-terminal IFITM2 (C-IFITM2) (1 μg) plasmid, respectively. Blank HEK293 cells served as control. Cells were collected 24 h post-transfection for total RNA extraction. IFN-β mRNA expression was detected via RT-qPCR. Data were represented as mean ± SDs of three independent experiments and were measured in technical duplication. Comparisons between groups were performed using the Student’s *t*-test. *** *p* < 0.001, ** *p* < 0.01, * *p* < 0.05.

To further determine the structural domains of IFITM2 that directly activates IFN-β and plays an antiviral role, we generated a series of truncated mutants N-terminal-IFITM2-1-85 aa and C-terminal-IFITM2-61-132 aa. These two plasmids were successfully expressed in HEK293 cells (Figure 9A). Next, we investigated the antiviral activity of the N-terminal and C-terminal of IFITM2 expression plasmids. Compared with the full-length plasmid (IFITM2-FL), the antiviral activity of the C-terminal plasmids was significantly weaker than that of the N-terminal plasmids. The viral copies number and TCID_50_ assay results supported this conclusion (Figure 9B,C). As IFITM2 exerts an antiviral effect associated with its ability to activate IFN-β, we similarly examined the transcription level of the IFN-β gene and found that the ability of the N-terminal and C-terminal of IFITM2 to activate IFN-β was significantly weaker than that of the full-length of IFITM2 (Figure 9D), which would explain why the truncated IFITM2 did not work as well as the full-length IFITM2 in the inhibition of the EMCV replication. These results suggest that the N-terminal domain of IFITM2 may play an important role in the antiviral activity and activation of IFN-β.

## 4. Discussion

IFN-α/β is a major contributor to antiviral defense, inducing the expression of hundreds of ISGs that encode antiviral proteins and modulators of the IFN induction and response pathways [19]. IFITM is one of these ISGs. IFITM proteins are the effectors of the initial host antiviral response and are engaged in a wide array of functions in the cell. The current study revealed that IFITM proteins potentiate antiviral responses by enhancing innate immune signaling pathways.

We examined the role of IFITM proteins in IFN production. Over-expression of IFITM1, IFITM2, and IFITM3 in HEK293 cells could significantly promote IFN-β production, both in mRNA transcription and protein expression levels. Additionally, cell culture supernatant could directly inhibit EMCV multiplication, which further proved that IFN-β was expressed and secreted in the culture supernatant.

In the past decades, mitochondrial antiviral immunity has been established with the identification of RIG-I/MDA5 and MAVS [20]. As EMCV has emerged as the prototypic MDA5-dependent virus [21,22], we detected whether IFITM2 protein expression affected MDA5 and MAVS mRNA and protein levels. Results showed that IFITM2 expression promoted MDA5 expression in HEK293 cells.

Many investigations have provided a detailed understanding of the intracellular antiviral signaling pathways. In our study, as IFITM2 showed stronger antiviral activity and the ability to activate IFN-β, we chose IFITM2 for further study. We verified that IFITM2 could bind to MDA5 in a co-immunoprecipitation assay. MDA5 was knocked down to determine the target molecule of IFITM2, and it was found that the ability of IFITM2 to activate IFN-β was significantly inhibited after interfering with MDA5 expression, suggesting that MDA5 may play an important role in the activation of the IFN-β signaling pathway by IFITM2.

The ability of a host to curb a viral infection is heavily reliant on the effectiveness of an initial antiviral innate immune response, resulting in the upregulation of IFN and, subsequentially, IFN-stimulated genes (ISGs). Recent research has uncovered a novel role for a handful of ISGs, some directly induced by IFN regulatory factor 3 in the absence of IFN itself. These ISGs, most with potent antiviral activity, can augment varying arms of the innate immune response to viral infection, thereby strengthening this response. These ISGs include protein kinase R (PKR), zinc-finger antiviral proteins (ZAPs), the DExD/H box helicase [23], and IFN-regulated members of the tripartite motif (TRIM)-containing families, such as TRIM21 [24,25] and TRIM56 [26,27,28]. In a recent study, we demonstrated that IFITM proteins could also promote the expression of IFN-β and can be used as an antiviral immune enhancer. This study will increase our understanding of IFITM proteins and innate immunity pathways.

**Figure 10:**
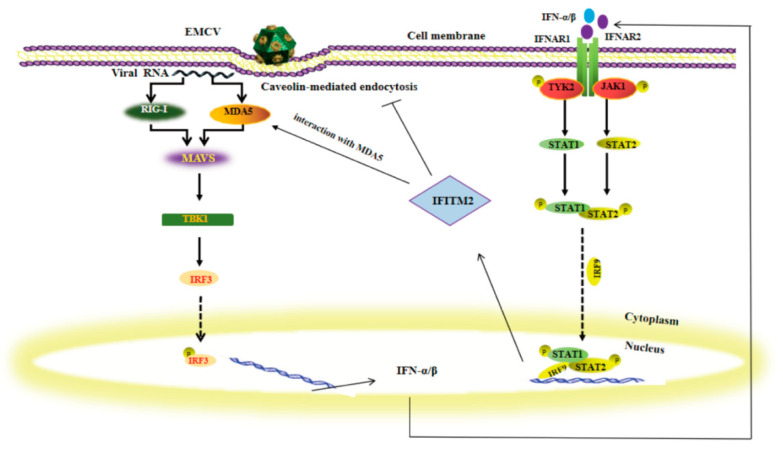
EMCV infected HEK293 cells via the caveolin-mediated endocytosis pathway. When the viral nucleic acid is exposed to the cytoplasm, it is recognized by pattern recognition receptor MDA5, which amplifies the signaling cascade and stimulates the host cell to produce type I IFN. Type I IFN binds to cell surface receptors and activates IFITM production via the JAK-STAT signaling pathway. IFITM presents an antiviral role in HEK293 cells during EMCV infection. Further study shows that IFITM2 could decrease Caveolin-1 expression and inhibit the virus entry process. Moreover, IFITM2 protein could increase MDA5 expression. In addition, there is an interaction between MDA5 and IFITM2. As IFITM2 is enhanced by IFN, these functions constitute a positive regulatory loop for IFN production.

Our study confirmed that IFITM proteins could directly influence the viral entry process by regulating the expression of endosomal compartments such as Rab5 and Caveolin-1 to inhibit viral replication or infection in HEK293 cells. In addition, IFITM proteins also enhance their antiviral effects by enhancing the IFN-β signaling pathway. Figure 10 is a model of the possible mechanism for IFN-β enhanced by IFITM2. As IFITM2 is induced by IFN, these functions constitute a positive regulatory loop circuit for IFN production.

As IFITM proteins are a kind of ISG produced by IFN and inhibit the proliferation of viruses in host cells, the authors believe that the regulation of immune response by IFITM and IFN-β constitutes a feedback loop. IFN-β stimulates the expression of IFITM proteins and the occurrence of antiviral immune responses, while IFITM proteins enhance the production of IFN-β and its antiviral response. This feedback regulation loop may play an important role in homeostasis and self-defense mechanisms in the body during inflammation, infection, and immune response.

## Data Availability

All available data are presented in the article.
